# Study on Rheological Properties of SBS/Crumb Rubber Modified Direct Coal Liquefaction Residue Asphalt Prepared Through an Extraction–Blending Process

**DOI:** 10.3390/ma19142940

**Published:** 2026-07-08

**Authors:** Yongxiang Li, Shizhong Mi, Chaoyang Guo, Jian Gao, Qi Qi, Yongjie Jia, Jing Li

**Affiliations:** 1 College of Energy and Transportation Engineering, Inner Mongolia Agricultural University, Hohhot 010018, China; jiayj@imau.edu.cn (Y.J.); li1728401678@163.com (J.L.); 2 Inner Mongolia Transportation Group Co., Ltd., Hohhot 010051, China; 2014021037@chd.edu.cn (S.M.); 13704733108@163.com (Q.Q.)

**Keywords:** direct coal liquefaction residue (DCLR), asphalt modification, SBS, crumb rubber, composite modification, rheological properties, high-temperature stability, low-temperature crack resistance, microstructure

## Abstract

To address the insufficient low-temperature performance of asphalt modified with direct coal liquefaction residue (DCLR), this study proposed a composite modification strategy based on an extraction–blending process using styrene–butadiene–styrene (SBS) and crumb rubber (CR). The high- and low-temperature rheological properties, phase morphology, and functional-group characteristics of DCLR-blended asphalt with different formulations were systematically evaluated using a dynamic shear rheometer (DSR), a bending beam rheometer (BBR), fluorescence microscopy (FM), and Fourier transform infrared spectroscopy (FTIR). The results demonstrate that the combined addition of SBS and crumb rubber significantly enhances the high-temperature stability and elastic response of the asphalt. Specifically, formulation 5# (8 wt.% SBS and 10 wt.% CR) maintained a rutting factor of 1.007 kPa at 82 °C, indicating superior high-temperature rutting resistance. Meanwhile, this formulation satisfied the Superpave low-temperature requirements at −18 °C, achieving a balanced improvement in both high- and low-temperature performance. Microstructural analysis suggests that an appropriate SBS/CR ratio contributes to the formation of a relatively continuous and uniformly distributed polymer-rich phase, whereas excessive modifier contents may lead to rubber agglomeration and phase-structure imbalance. FTIR results showed that the characteristic absorption peaks of the modified binders were generally consistent with those of the base asphalt, and no obvious new absorption bands were observed. This indicates that the extraction–blending process mainly involved physical blending, swelling, and phase interaction rather than the formation of new covalent functional groups. This study provides a technical reference for the high-value utilization of DCLR and the development of high-performance modified asphalt.

## 1. Introduction

Direct Coal Liquefaction Residue (DCLR), characterized by its complex composition and high carbon content, represents a significant solid waste whose high-value utilization is a crucial subject in the field of resource recycling. Given its chemical composition similarity to petroleum asphalt, containing substantial amounts of asphaltene and heavy oil components, DCLR is regarded as a potential road asphalt modifier and has garnered considerable attention in recent years [1,2,3].

Current research indicates that DCLR exerts a significant regulatory effect on the road performance of asphalt; however, its impact exhibits a distinct “dual nature” [4]. On one hand, the incorporation of DCLR can effectively enhance the high-temperature performance of asphalt and its mixtures. The addition of an appropriate amount (typically recommended not to exceed 10% of the base asphalt mass) can markedly increase the viscosity, hardness, and softening point of asphalt, while improving the rutting resistance and high-temperature stability of the mixture [5,6,7]. Dynamic mechanical analysis further confirms that, compared to conventional modified asphalts, DCLR-modified asphalt demonstrates a higher complex modulus, a lower phase angle, and superior elastic response and high-temperature deformation resistance [8,9]. Additionally, its resistance to moisture damage and cracking has also been improved to a certain extent [10,11]. On the other hand, the incorporation of DCLR typically impairs the low-temperature performance of asphalt, leading to a significant decrease in ductility, which constitutes the primary bottleneck limiting its large-scale application [12,13]. Studies suggest that the root of this performance contradiction lies in key components within DCLR—particularly tetrahydrofuran-insoluble substances and the high content of asphaltenes. While these components enhance the stiffness and thermal stability of the system, they disrupt the original colloidal structural homogeneity of the asphalt, thereby compromising its low-temperature flexibility [14,15].

To improve the balance between the high-temperature stability and low-temperature flexibility of DCLR-modified asphalt, composite modification has been widely investigated. Styrene–butadiene–styrene (SBS) copolymer is commonly used to improve the elastic recovery and deformation resistance of asphalt binders, whereas crumb rubber can contribute to flexibility and energy dissipation through swelling and interaction with light components in asphalt [16,17]. In addition, the use of recycled polymer and rubber wastes in bitumen modification has attracted increasing attention from the perspectives of resource recycling and sustainable pavement construction. Previous studies have shown that polymer waste and crumb rubber can improve the thermal stability, elasticity, and aging resistance of bituminous binders when appropriate dosages and processing conditions are adopted [11,18,19]. These findings indicate that polymer/rubber composite modification is a feasible strategy for improving asphalt performance while promoting the utilization of waste resources.

However, the performance of DCLR-modified asphalt remains strongly formulation-dependent. DCLR generally increases binder stiffness and improves rutting resistance, but excessive rigid fractions may impair low-temperature relaxation [20,21,22,23,24]. SBS and crumb rubber can compensate for this deficiency to some extent, but excessive polymer or rubber content may cause over-swelling, local agglomeration, phase imbalance, or reduced compatibility [25,26]. Therefore, the key issue is not merely whether DCLR, SBS, and crumb rubber can be combined, but how their proportions regulate the trade-off among high-temperature rutting resistance, low-temperature crack resistance, and phase-structure stability.

It should also be noted that aromatic-oil extraction and SBS/crumb rubber modification are not regarded in this study as completely new techniques. The extraction conditions were adopted from previous work and were used as a processing route to obtain DCLR solution asphalt for subsequent polymer modification. Accordingly, the main contribution of this study is not the proposal of a completely new extraction chemistry, but the evaluation of the formulation-dependent rheological behavior and phase morphology of DCLR-blended asphalt prepared through an extraction–blending route. In particular, attention was paid to whether an optimum SBS/crumb rubber ratio exists, whether a saturation or threshold effect can be observed, and how excessive modifier content affects the balance between high- and low-temperature performance.

To this end, DCLR-blended asphalt modified with SBS and crumb rubber was prepared through an extraction–blending process. The high-temperature rheological properties, low-temperature creep behavior, phase morphology, and functional-group characteristics were evaluated using dynamic shear rheometer (DSR), bending beam rheometer (BBR), fluorescence microscopy (FM), and Fourier transform infrared spectroscopy (FTIR) tests. The objective was to clarify the performance regulation characteristics of the DCLR–SBS–crumb rubber composite system and to provide a technical reference for the high-value utilization of DCLR in asphalt pavement materials.

## 2. Materials and Methods

### 2.1. Materials

The DCLR used in this study is a by-product from the direct coal liquefaction process of China Shenhua Coal-to-Liquid and Chemical Co., Ltd. (Beijing, China). Its main components and technical specifications are listed in Table 1 and Table 2, respectively. Aromatic oil (grade 4–26#) was selected, with its technical specifications provided in Table 3. The technical specifications of the chosen SBS are presented in Table 4, and those of the selected crumb rubber are detailed in Table 5.

The properties of the raw materials determine their respective functions in the extraction–blending process. DCLR contains a high proportion of asphaltene, pre-asphaltene, and THF-insoluble fractions, which can increase binder stiffness and contribute to high-temperature deformation resistance. However, these rigid fractions may also weaken low-temperature flexibility if they are directly incorporated into asphalt. Aromatic oil was therefore used as the extraction medium because of its high aromatic content and good compatibility with asphaltic components. It can dissolve part of the soluble organic fractions in DCLR and also act as a blending component in the subsequent modification process. SBS was selected because its linear block structure can improve the elastic response and deformation resistance of asphalt binders. Crumb rubber contains rubber hydrocarbons and carbon black, which can contribute to elasticity, flexibility, and waste-rubber utilization after swelling in the asphaltic phase. Therefore, the combined use of DCLR, aromatic oil, SBS, and crumb rubber was expected to improve the balance between high-temperature stability and low-temperature crack resistance.

### 2.2. Preparation of DCLR-Blended Asphalt

#### 2.2.1. Extraction of DCLR

Based on the “like-dissolves-like” principle, aromatic oil with a high aromatic content was used as the extraction medium for DCLR. The purpose of this process was to reduce the influence of tetrahydrofuran (THF)-insoluble matter and to obtain a liquid DCLR-derived phase suitable for subsequent blending with SBS and crumb rubber. Since no quantitative compositional analysis, such as SARA fractionation or GPC analysis, was conducted in the present study, the extraction effect was described mainly according to previous experimental results, the reported extraction procedure, and the FTIR functional-group characterization.

The DCLR extraction procedure was conducted as follows: Raw DCLR obtained from the direct coal liquefaction process and aromatic oil (grade 4–26#) were used as the extraction raw material and extraction medium, respectively. The solvent-to-DCLR mass ratio was selected as 5:4 according to preliminary extraction tests and the optimized procedure reported in Ref. [26]. Under this condition, the organic fractions in DCLR could be sufficiently dissolved, while the viscosity of the extracted liquid phase remained suitable for subsequent blending with SBS and crumb rubber. It should be noted that the term “selected” is used here instead of “optimal” because the extraction ratio was adopted from the previously optimized process rather than re-optimized in the present study. The measured aromatic oil and DCLR were placed in a reaction vessel. The mixture was subjected to moderate mechanical stirring at a constant temperature of 175 °C, maintained for 15 min to ensure sufficient contact and dissolution of the target components in DCLR by the solvent. Upon completion of the heating period, a hot filtration setup was used to promptly separate the liquid phase (primarily containing the dissolved asphaltene components and aromatic oil) from the solid residue (mainly comprising the undissolved THF-insoluble matter and ash). The resulting liquid phase, termed DCLR solution asphalt, served as the base component for the subsequent blending process.

According to the preliminary experimental investigations and orthogonal test validation reported in Ref. [26], this extraction route can remove approximately 40% of the THF-insoluble matter from DCLR and increase the relative proportion of soluble organic fractions in the liquid phase. In the present study, this previously reported extraction condition was adopted as a processing route for preparing DCLR solution asphalt. The obtained liquid phase was then used for subsequent blending with SBS and crumb rubber. Compared with conventional solvent extraction followed by solvent recovery, the use of aromatic oil as both the extraction medium and the blending component can simplify the preparation procedure.

#### 2.2.2. Preparation of DCLR-Blended Asphalt

The DCLR solution asphalt obtained from the extraction process was used as the base component for the preparation of DCLR-blended asphalt [28,29,30]. The DCLR solution asphalt was first heated to 180 °C and maintained under continuous mechanical shearing at 5000 r/min. The required amount of crumb rubber was then gradually added and sheared for 30 min. Subsequently, SBS was introduced into the blend, and high-speed shearing was continued for 60 min at the same temperature and shear rate. After shearing, the blend was kept at 175 °C for 45 min to complete the development process. The prepared DCLR-blended asphalt was then poured into molds for specimen preparation or stored for subsequent testing.

Based on preliminary single-factor tests and considerations of technical performance, material workability, and economy, SBS contents of 5 wt.%, 8 wt.%, and 10 wt.% and crumb rubber contents of 8 wt.%, 10 wt.%, and 12 wt.% were selected. All dosages were calculated by mass relative to the mass of DCLR solution asphalt. A full-factorial design was used to evaluate the effects of SBS and crumb rubber contents on the rheological properties and phase morphology of DCLR-blended asphalt. The selected dosage range was intended to cover relatively low, medium, and high polymer/rubber contents, thereby allowing the influence of modifier dosage and possible threshold behavior to be examined. The experimental groups are listed in Table 6.

From an environmental and practical perspective, the preparation temperature used in this study (175–180 °C) is comparable to that commonly adopted for polymer-modified asphalt binders. In addition, aromatic oil served not only as the extraction medium for DCLR but also as a blending component in the subsequent asphalt modification process. Therefore, a separate solvent recovery step was not required in this preparation route, which may simplify the process compared with conventional solvent extraction. Nevertheless, a complete environmental assessment, economic evaluation, and scale-up analysis were beyond the scope of the present study. These aspects should be further investigated before large-scale engineering application.

### 2.3. Experimental Methods

#### 2.3.1. Dynamic Shear Rheometer (DSR) Test

The high-temperature rheological properties of various DCLR-blended asphalt formulations were systematically evaluated using a DHR-I DSR from TA Instruments, (New Castle, DE, USA), through strain sweep and temperature sweep tests.

Rheological studies of asphalt are typically conducted within the linear viscoelastic region to ensure data reliability. Therefore, determining this region for the asphalt is a prerequisite. Strain sweep tests were performed to establish the linear viscoelastic range for subsequent testing. Relevant studies indicate that the linear viscoelastic region narrows at lower temperatures [31]. Considering the focus on high-temperature performance, a frequency of 10 rad/s was used. This frequency was selected because it is commonly used for comparative evaluation of the high-temperature rheological response and rutting-related parameters of asphalt binders. The purpose of this study was to compare the formulation-dependent variation in rheological behavior under a consistent loading condition rather than to construct a full frequency-dependent master curve. It should be acknowledged that asphalt binders exhibit frequency-dependent viscoelastic behavior; therefore, frequency sweep tests and master-curve analysis will be considered in future work to provide a more comprehensive description of the viscoelastic characteristics of DCLR-blended asphalt. The limit of the linear region was defined as the strain level at which the complex shear modulus (G*) decreased by 10% at a test temperature of 46 °C. Strain sweep tests were conducted at five temperatures: 46 °C, 52 °C, 58 °C, 64 °C, and 70 °C, with a strain range of 0.1% to 100% and a fixed frequency of 10 rad/s.

Temperature sweep tests were performed on the different DCLR-blended asphalt formulations. A 25 mm parallel plate geometry with a 1 mm gap was used. The tests were conducted under a strain level of 10% and a loading frequency of 10 rad/s, across a temperature range from 46 °C to 82 °C at 6 °C intervals. This procedure provided the variation trends of the G* and phase angle (δ) with temperature for each DCLR-blended asphalt.

The strain level of 10% used in the temperature sweep test was lower than the minimum critical strain obtained from the strain sweep test. Therefore, the temperature sweep measurements were conducted within the linear viscoelastic region for all investigated formulations. However, because the critical strain of formulation 1# was 14.9%, the selected strain was relatively close to its linear viscoelastic boundary. Accordingly, the results of 1# were interpreted with caution, and the main conclusions were drawn based on the overall trends among different formulations rather than on a single data point.

#### 2.3.2. Bending Beam Rheometer (BBR) Test

According to the specifications of the U.S. Strategic Highway Research Program (SHRP), the BBR was employed to evaluate the low-temperature performance of the asphalt, using the creep stiffness (S) and the creep rate (m-value) as the key indicators. Following the Superpave system requirements, the test results at a loading time of 60 s were taken, with the qualification criteria being S ≤ 300 MPa and m ≥ 0.3. In this study, BBR tests were conducted at temperatures of −12 °C, −18 °C, and −24 °C to measure the S and m-values of each asphalt binder sample, thereby assessing their low-temperature rheological behavior. The values of S and m were calculated using the following formulas, respectively:

(1) $$S(t)=\frac{PL^{3}}{4bh^{3}\delta (t)}$$

(2) $$m=\frac{lg(s)}{lg(t)}$$

where

P—constant load;

L—beam span length (mm);

b—beam width (mm);

h—beam height (mm);

δ(t)—mid-span deflection at loading time t.

#### 2.3.3. Fluorescence Microscopy (FM) Test

A Leica DM2700M upright metallurgical microscope equipped with a fluorescence light source and corresponding filter sets was employed to observe the micromorphology and phase structure of the DCLR-blended asphalt. The observation conditions were set at an excitation wavelength of 450–490 nm and a magnification of 200×. Under fluorescence mode, the SBS phase (bright white) was distinguished from the asphalt matrix (dark background) to analyze the dispersion state of SBS and crumb rubber, the distribution of the polymer-rich phase, and the phase morphology of the DCLR-blended asphalt.

#### 2.3.4. Fourier Transform Infrared Spectroscopy (FTIR) Test

Fourier transform infrared spectroscopy (FTIR) was used to characterize the functional groups of the 90# base asphalt and the DCLR-blended asphalt binders. The spectra were collected over a wavenumber range of 4000–600 cm^−1^. The FTIR results were used to identify the characteristic absorption bands of the asphalt binders and to examine whether new functional groups were formed during the extraction–blending modification process.

#### 2.3.5. Reproducibility and Data Processing

To ensure the reliability of the experimental results, repeated measurements were conducted for the rheological tests. For the DSR strain sweep and temperature sweep tests, at least two independent specimens were tested for each asphalt formulation, and the average values were used for subsequent analysis. For the BBR test, three beam specimens were tested for each formulation at each temperature, and the average creep stiffness and m-value at 60 s were reported. The error bars in the BBR results represent the standard deviation of the three replicate beam specimens. When abnormal values caused by specimen preparation defects or instrument instability were observed, the test was repeated to ensure data reliability.

For fluorescence microscopy observation, at least three different fields of view were examined for each selected asphalt sample. Representative images were selected to describe the phase morphology and dispersion characteristics of the polymer-rich phase. Since FM was mainly used for qualitative microstructural observation in this study, no statistical significance test was performed for the microscopy results.

The variability of the rheological measurements was assessed by repeated testing. The differences among repeated values were within an acceptable range and did not affect the main trends discussed in this study. Therefore, the reported results were considered reliable for comparing the effects of SBS and crumb rubber contents on the rheological properties and phase morphology of DCLR-blended asphalt. It should be noted that the statistical analysis in this study was mainly used to verify repeatability, while more detailed statistical significance analysis will be considered in future work.

## 3. Results

### 3.1. Analysis of Strain Sweep Tests

**Figure 1:**
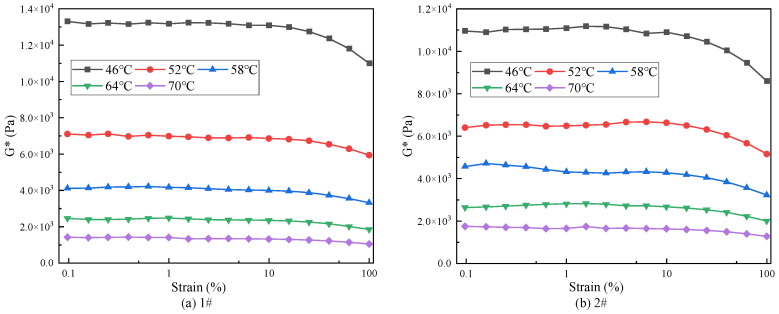

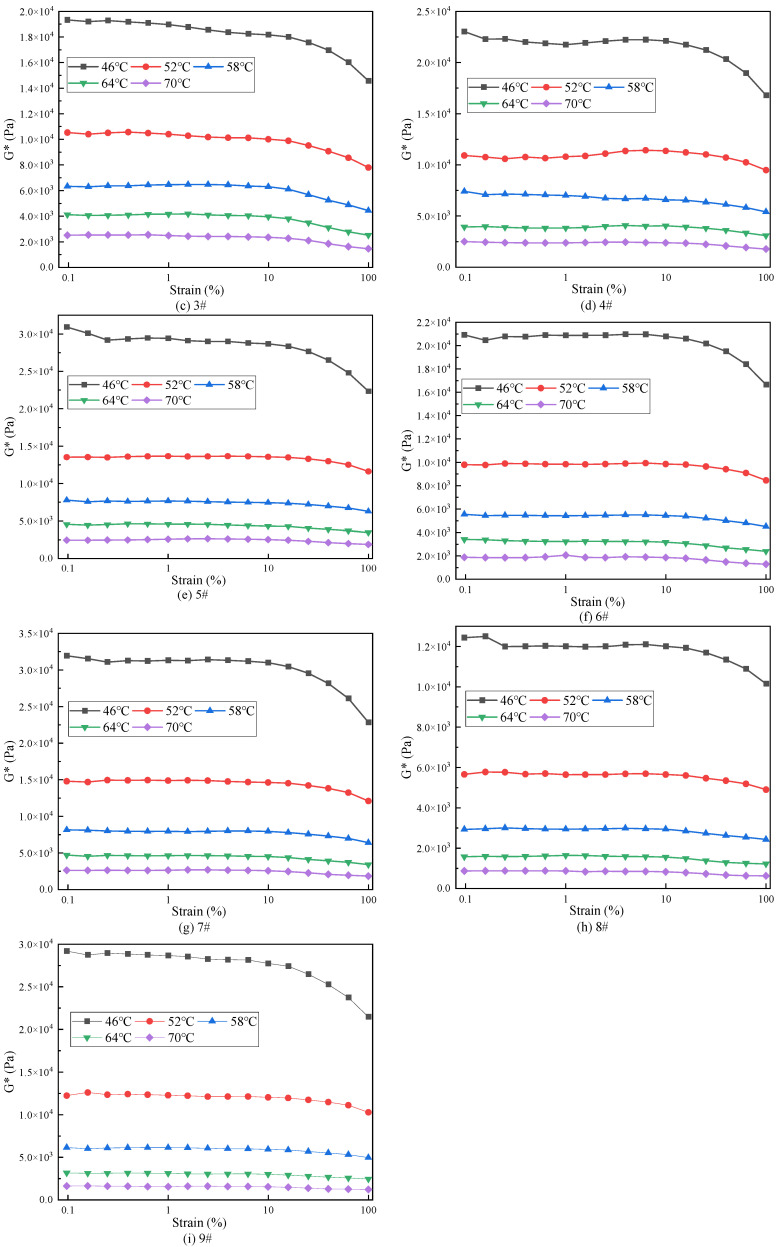
Strain sweep results of DCLR-blended asphalt with different formulations.

Figure 1 presents the strain sweep results for the DCLR-blended asphalts with different formulations. At 52 °C, 58 °C, 64 °C, and 70 °C, the strain sweep curves generally exhibited a gentle overall variation. Within this temperature range, the DCLR-blended asphalts demonstrated sustained linear viscoelastic rheological behavior across the applied strain range of 0.1% to 100%. The characteristic responses of the nine asphalt groups shown in Figure 1 were consistent, with an overall trend of decreasing G* as strain increased. However, the most pronounced change in G* occurred at 46 °C. Within a relatively low strain range, the decrease in G* did not exceed 10% of its maximum value. As the strain increased beyond a certain threshold, the rate of decrease in G* became significantly more pronounced, indicating a transition of the DCLR-blended asphalt from the linear viscoelastic strain region to the non-linear viscoelastic strain region.

The critical strain is defined as the strain value at which the G* decreases to 90% of its initial linear value. This value, determined from the relationship curve between G* and strain obtained in the strain sweep test, serves as the key boundary distinguishing the material’s linear and non-linear viscoelastic behavior. Its magnitude reflects the material’s maximum deformation capacity to maintain structural integrity and a linear response under shear. Table 7 lists the critical strain limits within the linear viscoelastic region for each asphalt group. Based on the method for determining the linear viscoelastic range, the respective ranges for the different DCLR-blended asphalts were established. The incorporation of SBS and crumb rubber, coupled with an increasing dosage, initially expanded and then subsequently reduced the linear viscoelastic range of the DCLR-blended asphalt. This pattern may be associated with excessive modifier content, which could disturb the dispersion state of the polymer/rubber phase and reduce the stability of the linear viscoelastic response. Identifying this regularity laid the foundation for selecting parameters in subsequent temperature sweep tests. Among the samples, the 1# DCLR-blended asphalt exhibited the smallest linear range (0–14.9%), while the 5# DCLR-blended asphalt showed the largest (0–53.1%). Consequently, a strain level of 10% was selected for all subsequent tests. Although the selected strain level was lower than the minimum critical strain obtained from the strain sweep tests, it was relatively close to the LVE boundary of formulation 1#, whose critical strain was 14.9%. Therefore, the results of formulation 1# were interpreted with caution. The main conclusions of this study were drawn based on the overall variation trends among different formulations rather than on the response of a single sample near the LVE boundary.

### 3.2. Analysis of Temperature Sweep Tests

**Figure 2:**
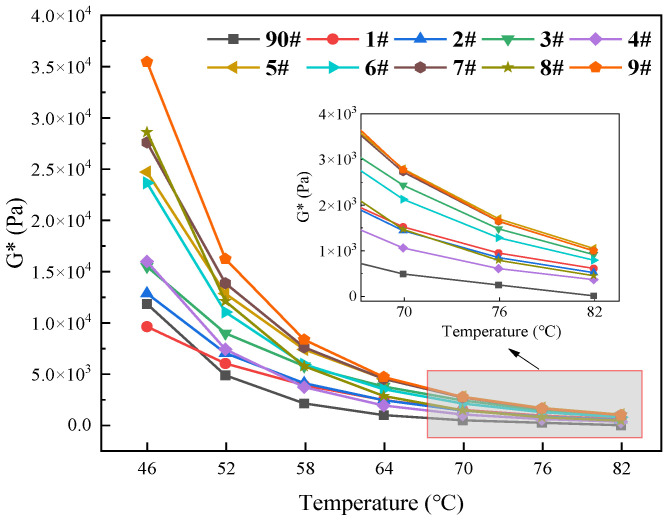
Complex shear modulus of DCLR-blended asphalt with different formulations.

The G* is defined as the ratio of the maximum shear stress (τ_max_) to the maximum shear strain (γ_max_), reflecting the ability of an asphalt sample to resist deformation under repeated shear loading. Figure 2 presents the G* values of different DCLR-blended asphalt formulations. At 46 °C, the G* of the 1# DCLR-blended asphalt was 18.6% lower than that of the 90# base asphalt. At all other test temperatures, the G* of the nine blended asphalts exceeded that of the base asphalt but decreased with rising temperature. This decline occurs because increased thermal motion of asphalt molecules at higher temperatures weakens intermolecular attraction, softening the asphalt and reducing its G*, thereby diminishing its deformation resistance.

Notably, the G* of the 6# formulation was 4.3% lower than that of the 5# formulation. This result should not be interpreted as direct evidence that the colloidal system had reached a complete equilibrium state. A more cautious explanation is that the increase in crumb rubber content from 10 wt.% to 12 wt.% may have moved the binder from a reinforcement-dominated stage to a modifier-saturation stage. In this stage, excessive rubber content may induce over-swelling, incomplete dispersion, localized phase imbalance, or partial disruption of the polymer-rich phase, thereby reducing the effective stress-transfer capacity of the modified binder. Therefore, the lower G* of 6# indicates that further increasing modifier content does not necessarily lead to continuous improvement in high-temperature stiffness.

**Figure 3:**
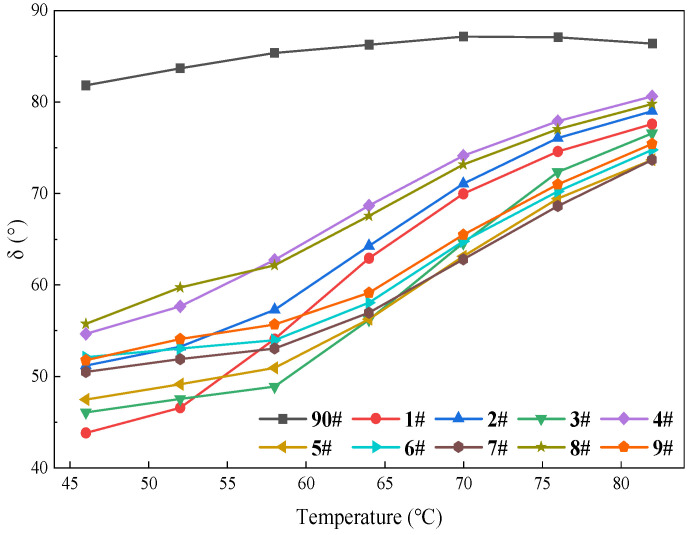
Phase angle of DCLR-blended asphalt with different formulations.

The δ quantifies the viscoelastic character of asphalt, representing the proportion between the viscous (irrecoverable) and elastic (recoverable) components within the binder. Figure 3 presents the phase angle of the different DCLR-blended asphalt formulations. For the 1#–9# DCLR-blended asphalts, the phase angle ranged between 40° and 80° across the temperature span of 46 °C to 82 °C. In contrast, the phase angle of the 90# base asphalt remained within a higher range of 80° to 88°.

The consistently lower phase angles of all DCLR-blended asphalts compared with the base asphalt indicate that the elastic contribution of the binder was enhanced after composite modification. This behavior may be associated with the swelling of SBS and crumb rubber and the formation of a relatively continuous polymer-rich phase in the DCLR-blended asphalt. As a result, the modified binders exhibited stronger resistance to viscous flow at elevated temperatures than the 90# base asphalt.

At the highest test temperature of 82 °C, formulation 5# exhibited the lowest phase angle among the investigated formulations. This indicates that formulation 5# had a relatively stronger elastic response and better phase-structure stability under high-temperature conditions. This result is consistent with the rutting factor analysis, suggesting that an appropriate SBS/CR ratio was beneficial for improving the high-temperature deformation resistance of DCLR-blended asphalt.

**Figure 4:**
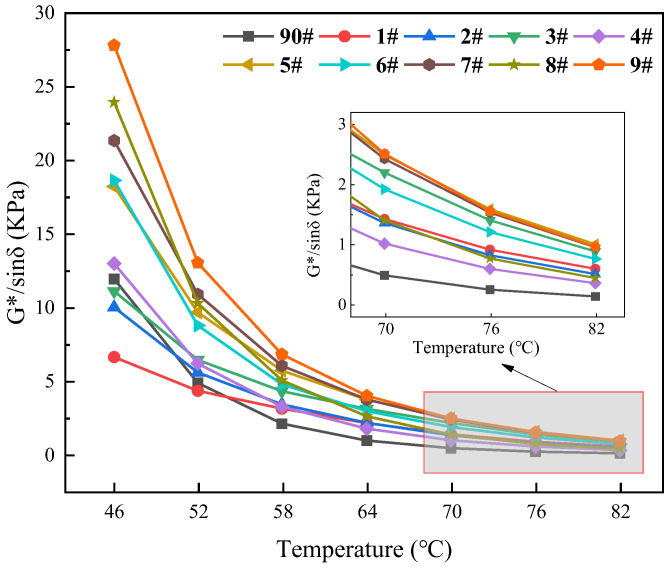
Rutting factor of DCLR-blended asphalt with different formulations.

The rutting factor (G*/sin δ) is a critical parameter for evaluating the high-temperature performance and rutting resistance of asphalt binders. Figure 4 presents the rutting factor for the different DCLR-blended asphalt formulations. While the base asphalt met the specification requirement at 64 °C, all nine DCLR-blended asphalts satisfied the requirement at 70 °C, representing an enhancement of one performance grade in high-temperature capability compared to the 90# base asphalt. Notably, the 5# DCLR-blended asphalt met the requirement even at 82 °C, with a G*/sin δ value of 1.007 kPa. Analysis based on the rutting factor indicates that formulation 5# possessed superior rutting resistance and high-temperature stability compared with the other blends, suggesting that the SBS/CR ratio in this formulation was more favorable for enhancing the elastic response and deformation resistance of DCLR-blended asphalt.

A comprehensive analysis of the rutting factor results yields the following high-temperature performance ranking: 5# > 9# > 7# > 6# > 4# > 8# > 3# > 2# > 1# > 90# base asphalt. It should be noted that this ranking only reflects high-temperature rutting resistance and should not be regarded as an overall performance ranking. The overall performance of DCLR-blended asphalt should be evaluated by considering both high-temperature rutting resistance and low-temperature cracking resistance.

### 3.3. Analysis of High-Temperature Susceptibility

Temperature susceptibility refers to the degree to which the viscosity and plasticity of asphalt are affected by temperature changes. The GTS index (Temperature Susceptibility Index based on Complex Shear Modulus) can reflect the influence of temperature variations between 46 °C and 82 °C on asphalt performance, providing a more realistic and comprehensive description of the temperature susceptibility of the DCLR-blended asphalts.

**Figure 5:**
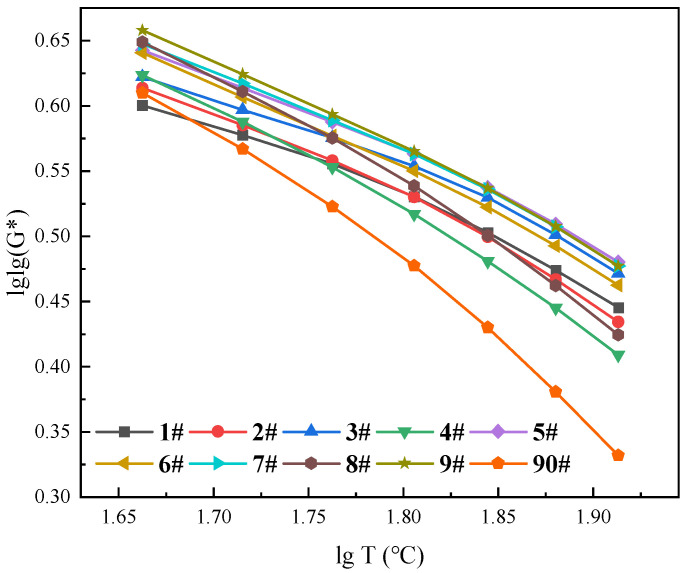
High-temperature susceptibility fitting curves of DCLR-blended asphalt with different formulations.

The temperature susceptibility of the DCLR-blended asphalts is described based on the GTS index values, with tests conducted from 46 °C to 82 °C. Using the logarithm of temperature (lg T) as the abscissa and the double logarithm of the complex modulus (lg lg G*) as the ordinate, the slope of the regression equation represents the GTS value. The high-temperature susceptibility fitting curves for the different DCLR-blended asphalt formulations are shown in Figure 5, and the corresponding fitting results are presented in Table 8.

The GTS value reflects the sensitivity of the complex shear modulus to temperature variation within the investigated temperature range. As shown in Table 8 and Figure 5, the R^2^ values of all fitted curves were close to 1.0, indicating that the fitting quality was acceptable for all formulations. Therefore, formulations 1#–3# should not be excluded solely because their R^2^ values were slightly lower than that of the base asphalt. In the revised analysis, all formulations were retained for comparison, and the GTS index was used only as an indicator of the temperature sensitivity of G*. A lower absolute GTS value indicates that binder stiffness is less sensitive to temperature variation, but it should not be used alone to determine the internal stability of the modified binder. Compared with the 90# base asphalt, all DCLR-blended asphalt formulations showed lower |GTS| values, indicating that the composite modification reduced the high-temperature susceptibility of the binder to different extents. Although formulation 3# exhibited the lowest |GTS| value, its rutting resistance was not as favorable as that of formulation 5#. Therefore, high-temperature performance should be evaluated by considering both rutting factor and temperature susceptibility. From this perspective, formulation 5# exhibited a better balance between high-temperature rutting resistance and temperature stability.

### 3.4. Analysis of Creep Stiffness and Creep Rate

**Figure 6:**
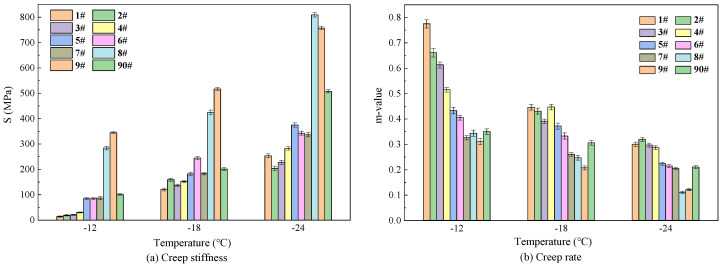
Creep stiffness and the creep rate of DCLR-blended asphalt with different formulations.

Figure 6 presents the S and m-value of the different DCLR-blended asphalt formulations. Under varying temperature conditions, the creep stiffness of each DCLR-blended asphalt group gradually increased as the temperature decreased, while the creep rate showed the opposite trend. The 90# base asphalt barely met the specification requirements at −18 °C.

At −24 °C, the overall performance of all DCLR-blended asphalts, including the 5# formulation, declined, with most failing to meet the index requirements, indicating a susceptibility to low-temperature cracking. The creep rate of all groups decreased with lower temperature. At −24 °C, only the 1# and 2# DCLR-blended asphalts satisfied the m-value requirement, which may be associated with the relatively higher availability of flexible fractions in these formulations, as inferred from the raw-material characteristics and previous studies. At −18 °C, only the 7#, 8#, and 9# blended asphalts failed to meet the requirements.

The BBR results in this section describe the absolute low-temperature cracking resistance of the binder at a given test temperature. Specifically, lower creep stiffness and higher m-value indicate better stress relaxation ability under that specific low-temperature condition. A comprehensive analysis indicates that formulations 9#, 8#, and 7# exhibited relatively poor low-temperature performance. This may be associated with excessive SBS and crumb rubber contents, which could restrict molecular mobility and reduce stress relaxation ability at low temperatures. In contrast, formulations 1#, 2#, and 3# showed better absolute low-temperature resistance because their relatively lower modifier contents were more favorable for maintaining flexibility and stress relaxation ability. However, these formulations did not show the best high-temperature rutting resistance. Therefore, they should not be regarded as the optimum formulations for overall pavement performance.

Taking −18 °C as the evaluation temperature, formulations 1# to 6# satisfied the Superpave low-temperature requirements and exhibited better low-temperature performance than the 90# base asphalt based on creep stiffness and m-value.

### 3.5. Analysis of Low-Temperature Susceptibility

The S of the asphalt increased while the m-value decreased, and this inverse relationship was consistently observed across all asphalt groups tested. The temperature susceptibility of the asphalt was characterized by analyzing the relationship between the S, measured using a BBR, and temperature. Regression analysis was subsequently performed on the S versus temperature for the nine asphalt blends.

(3) $$lgS=-S_{AS}\cdot T+C$$

where S represents the creep stiffness, S_AS_ is the creep stiffness index, T denotes temperature, and C is a constant.

The S_AS_ index characterizes the rate of change of the logarithm of creep stiffness (lg S) with temperature for each asphalt group, representing its temperature susceptibility. It should be distinguished from the absolute low-temperature cracking resistance discussed in Section 3.4. The SAS index reflects the sensitivity of creep stiffness to temperature variation in the low-temperature range, rather than the cracking resistance at a single temperature. Therefore, formulations 1#–3# may exhibit better absolute low-temperature resistance at specific temperatures, whereas formulations 5# and 6# may show lower temperature susceptibility and better stability against temperature variation. The low-temperature susceptibility fitting results for the ten asphalt groups are presented in Table 9. Overall, the S_AS_ values gradually decreased, indicating a reduction in the creep stiffness index of the asphalt, a diminished influence from low-temperature environmental conditions, and a consequent gradual increase in low-temperature stability.

Using the low-temperature susceptibility of the 90# base asphalt as a benchmark, the 5#, 6#, 7#, 8#, and 9# DCLR-blended asphalts exhibited relatively smaller S_AS_ values. This suggests their lower sensitivity to low temperatures and relatively better stability under such conditions. However, considering the performance analysis, the 7#, 8#, and 9# DCLR-blended asphalts did not meet the performance requirements at −18 °C. Therefore, the 5# and 6# blends demonstrate relatively superior low-temperature stability amidst temperature variations in the low-temperature region.

### 3.6. Microstructural Characterization

**Figure 7:**
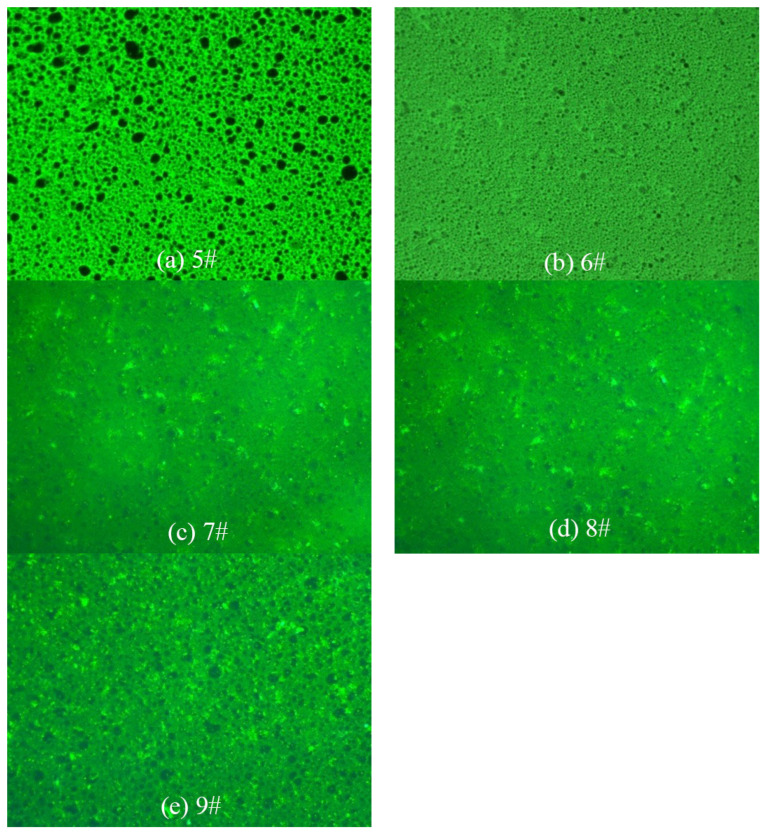
Fluorescence microscopy results.

Fluorescence microscopy was used to observe the phase morphology of the DCLR-blended asphalt binders. Under fluorescence mode, the polymer-rich phase could be distinguished from the asphalt-rich phase, allowing the dispersion state of SBS and crumb rubber to be qualitatively evaluated. It should be noted that FM provides two-dimensional morphological information; therefore, the observed structure is described in this study as a network-like polymer-rich phase rather than direct evidence of a three-dimensional interpenetrating network. The FM experimental results are shown in Figure 7.

For the 5#, a relatively continuous and uniformly distributed polymer-rich phase was observed. No obvious large-scale agglomeration of crumb rubber was detected, indicating that SBS and crumb rubber were adequately swollen and dispersed within the DCLR-blended asphalt. This morphology is consistent with the rheological results, in which the 5# exhibited the largest linear viscoelastic strain range, the lowest phase angle at high temperature, and the highest rutting factor among the investigated formulations. These results suggest that an appropriate proportion of SBS and crumb rubber can improve the structural integrity of the binder and provide an effective pathway for stress transfer under high-temperature loading.

In contrast, 7#, 8#, and 9# showed less uniform phase distribution, and localized agglomerates were observed in the fluorescence images. This phenomenon indicates that excessive SBS and crumb rubber contents may exceed the capacity of the DCLR-blended asphalt system to maintain a stable colloidal structure. Under such conditions, part of the rubber particles may not be fully incorporated into the polymer-rich phase, resulting in a less homogeneous microstructure. This structural imbalance is consistent with the deterioration in low-temperature performance observed in the BBR test.

The rheology–morphology relationship of the DCLR–SBS–CR system can be interpreted from the perspective of phase compatibility and structural balance. DCLR contains aromatic/asphaltene-rich fractions that may contribute to stiffness and high-temperature deformation resistance. Crumb rubber may improve elasticity and flexibility after swelling, while SBS contributes to the formation of a polymer-rich phase that enhances the elastic response of the binder. When the proportions of SBS and CR are appropriate, these components may form a more compatible multiphase structure. However, excessive modifier content may disturb phase distribution and induce local agglomeration, thereby weakening the expected improvement in rheological performance.

Therefore, the superior performance of formulation 5# may be associated not only with the presence of SBS and CR, but also with the balanced interaction among DCLR composition, polymer swelling, rubber dispersion, and phase compatibility. This interpretation provides a more reasonable link between the microscopic morphology and macroscopic rheological behavior of DCLR-blended asphalt.

More importantly, the identification of formulation 5# should not be regarded merely as an empirical optimization result. Within the investigated dosage range, the results suggest that an SBS/CR ratio of 8:10 provided a better balance between high-temperature rutting resistance and low-temperature crack resistance. When the crumb rubber content was further increased to 12 wt.% or when the total modifier content became excessive, the high-temperature stiffness did not improve continuously and the low-temperature performance tended to deteriorate. These results indicate the existence of a modifier-saturation or threshold effect in the DCLR-blended asphalt system. Therefore, the performance of DCLR-blended asphalt is governed by the balance among DCLR composition, polymer-rich phase formation, crumb rubber swelling and dispersion, and phase compatibility, rather than by modifier dosage alone.

It should be emphasized that the FM results provide qualitative two-dimensional morphological evidence. Therefore, the observed phase morphology was used to support the rheological trends, rather than to directly prove a three-dimensional network structure or molecular-level interaction.

The above results are generally consistent with previous studies on DCLR-modified asphalt and SBS/crumb rubber composite modified asphalt. Previous studies have reported that DCLR can improve the high-temperature stiffness and rutting resistance of asphalt binders, but excessive rigid fractions may adversely affect low-temperature relaxation. Studies on SBS and crumb rubber composite modification have also shown that polymer and rubber modifiers can enhance the elastic response of asphalt, whereas excessive modifier contents may lead to swelling imbalance, local agglomeration, or reduced phase compatibility. In the present study, formulation 5# showed a better balance between rutting resistance and low-temperature cracking resistance, while formulations with higher modifier contents did not show continuous performance improvement. This phenomenon indicates that the performance of DCLR-blended asphalt is controlled not only by the total modifier dosage, but also by the compatibility and structural balance among DCLR-derived fractions, SBS, and crumb rubber. Therefore, the observed rheological trends and FM morphology support the existence of a formulation-dependent saturation or threshold effect in the investigated DCLR–SBS–CR system.

### 3.7. Fourier Transform Infrared Spectroscopy Analysis

**Figure 8:**
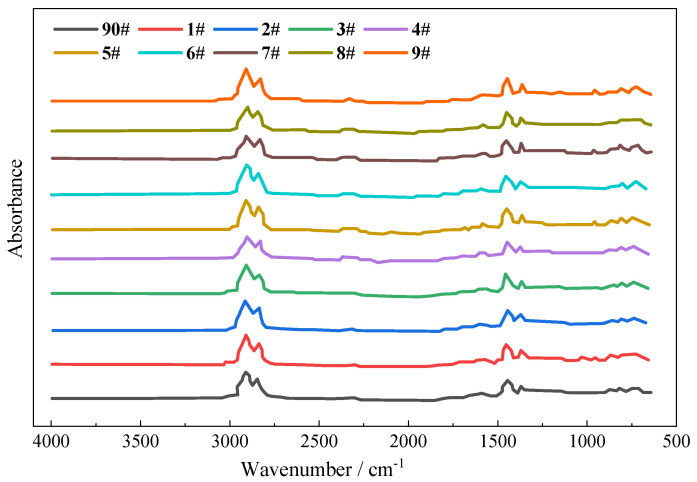
FTIR spectra of 90# base asphalt and DCLR-blended asphalt binders with different SBS/CR formulations.

Figure 8 shows the FTIR spectra of the 90# base asphalt and the DCLR-blended asphalt binders with different SBS/CR formulations. The spectra of all binders exhibited similar characteristic absorption bands, indicating that the main functional groups of the asphalt binders were generally retained after the extraction–blending modification process. The absorption peaks near 2920 cm^−1^ and 2850 cm^−1^ were attributed to the stretching vibration of aliphatic —CH_2_ and —CH_3_ groups. The peak near 1600 cm^−1^ was associated with the skeletal vibration of aromatic C=C bonds. The bands around 1455 cm^−1^ and 1375 cm^−1^ corresponded to the bending vibration of —CH_2_ and —CH_3_ groups, while the absorption bands in the range of 870–720 cm^−1^ were related to the out-of-plane bending vibration of aromatic C—H bonds.

No obvious new absorption bands or significant peak shifts were observed in the spectra of the DCLR-blended asphalt binders compared with the 90# base asphalt. This indicates that the incorporation of DCLR-derived fractions, SBS, and crumb rubber did not produce detectable new covalent functional groups under the investigated preparation conditions. Therefore, the performance improvement of the DCLR-blended asphalt was more likely associated with physical blending, swelling of the polymer/rubber phase, and phase-structure adjustment rather than a distinct chemical reaction.

It should be noted that the FTIR spectra were mainly used for qualitative functional-group identification. Since the spectra in Figure 8 are vertically offset for clarity, the differences in peak intensity should be interpreted cautiously. Nevertheless, the similarity of the characteristic peak positions among all formulations supports the interpretation that the extraction–blending process mainly changed the physical dispersion and phase morphology of the binder system, which is consistent with the FM observations and rheological results.

## 4. Conclusions

In this study, DCLR-blended asphalt modified with SBS and crumb rubber was prepared through an extraction–blending process. The effects of SBS and crumb rubber contents on the rheological properties and phase morphology of the modified binders were evaluated using DSR, BBR, and FM tests. The following conclusions can be drawn:

(1) The extraction–blending process combined DCLR extraction with subsequent polymer modification. Compared with 90# base asphalt, the DCLR-blended asphalt binders exhibited higher complex shear modulus, lower phase angle, and improved rutting resistance at elevated temperatures, indicating that the composite modification enhanced the elastic response and high-temperature deformation resistance of the binder.

(2) The formulation had a significant influence on the rheological behavior of DCLR-blended asphalt. Formulation 5#, containing 8 wt.% SBS and 10 wt.% CR, showed the best balance between high-temperature rutting resistance and low-temperature crack resistance among the investigated formulations. It maintained a rutting factor of 1.007 kPa at 82 °C and satisfied the Superpave low-temperature requirements at −18 °C.

(3) The FM observations showed that 5# exhibited a relatively uniform and continuous polymer-rich phase, whereas excessive SBS and crumb rubber contents led to local agglomeration and phase-structure imbalance. The microstructural observations were consistent with the rheological results, suggesting that the performance improvement was closely related to polymer swelling, rubber dispersion, and the compatibility of the DCLR-rich phase.

(4) The FTIR spectra showed that the characteristic absorption peaks of the DCLR-blended asphalt binders were generally consistent with those of the 90# base asphalt, and no obvious new absorption bands were observed. This indicates that the extraction–blending modification process mainly involved physical blending, polymer/rubber swelling, and phase-structure adjustment rather than the formation of new covalent functional groups. Together with the FM and rheological results, the performance of DCLR-blended asphalt was found to be governed by the balance among DCLR composition, polymer-rich phase formation, crumb rubber swelling, and phase compatibility. Further studies involving quantitative compositional analysis, such as SARA fractionation or GPC analysis, and mechanical validation are still needed to clarify the molecular-level interaction mechanism of this composite system.

## Figures and Tables

**Table 1:** Main components of DCLR.

Component	Heavy Oil	Asphaltene	Pre-Asphaltene	THF-Insoluble Substances
Content (%)	10~20	22~26	15~25	43~46

**Table 2:** Technical specifications of DCLR.

Technical Indicator	Density (g/cm^3^)	Softening Point (°C)	Penetration at 25 °C (0.1 mm)	Ductility at 10 °C (cm)
Test result	1.24	175	4.8	0.5

**Table 3:** Technical specifications of aromatic oil.

Technical Indicator	Kinematic Viscosity (m^2^/s)	Flash Point (°C)	Ash Content (%)	Density (g/cm^3^)	Aromatic Content (%)	Appearance
Test result	28	229	0.01	0.902	80	Yellowish-green

**Table 4:** Basic performance indicators of SBS.

Grade	Structure	Block Ratio (S/B)	Volatile Content (%)	Melt Flow Rate (g/10 min)	Ash Content (%)	Tensile Strength (MPa)	Elongation at Break (%)
YH-791	Linear	30/70	≤0.44	0.09	≤0.2	≥21.5	≥733

**Table 5:** Physical and chemical properties of crumb rubber [27].

Test Item	Technical Requirement	Test Result
Sieve residue (%)	<10	8
Relative density (g/cm^3^)	1.10~1.30	1.20
Moisture content (%)	<1.0	0.55
Iron content (%)	<0.03	0.02
Fiber content (%)	<1.0	0.04
Ash content (%)	≤8	6
Acetone extract (%)	≤16	11
Carbon black content (%)	≥28	32
Rubber hydrocarbon content (%)	≥48	56
Solubility (%)	≥16	18

**Table 6:** Experimental groups of DCLR-blended asphalt with different formulations.

Group No.	SBS Content (wt.%)	Crumb Rubber Content (wt.%)
1#	5	8
2#	5	10
3#	5	12
4#	8	8
5#	8	10
6#	8	12
7#	10	8
8#	10	10
9#	10	12

Note: 1–9# denote Sample No. 1–9.

**Table 7:** Critical strain of DCLR-blended asphalt with different formulations.

Group No.	1#	2#	3#	4#	5#	6#	7#	8#	9#
Strain (%)	14.9	26.4	52.6	44.2	53.1	33.6	32.5	28.7	38.8

**Table 8:** High-temperature susceptibility fitting results of DCLR-blended asphalt with different formulations.

Group No.	GTS	C	R^2^	|GTS|
1#	−0.61	1.63	0.981	0.61
2#	−0.7	1.8	0.985	0.7
3#	−0.5	1.6	0.981	0.5
4#	−0.85	2	0.992	0.85
5#	−0.63	1.7	0.991	0.63
6#	−0.69	1.8	0.994	0.69
7#	−0.67	1.76	0.992	0.67
8#	−0.89	2.14	0.991	0.89
9#	−0.71	1.8	0.995	0.71
90#	−1.1	2.46	0.988	1.1

**Table 9:** Low-temperature susceptibility fitting results of DCLR-blended asphalt with different formulations.

Asphalt Type	R^2^	S_AS_	C
1#	0.98	0.105	0.03
2#	0.98	0.0878	0.36
3#	0.98	0.0872	0.37
4#	0.98	0.08	0.57
5#	0.99	0.053	1.28
6#	0.98	0.05	1.37
7#	0.99	0.049	1.36
8#	0.98	0.037	1.98
9#	0.999	0.028	2.2
90#	0.992	0.0584	1.29

## Data Availability

The original contributions presented in this study are included in the article. Further inquiries can be directed to the corresponding author.

## References

[1] Zhang Y. (2021). Study on the Process and Performance of Coal Liquefaction Asphalt Modified Petroleum Asphalt. Master’s Thesis.

[2] Ji J., Wang D., Shi Y., Xu S., Suo Z. (2016). Performance Evaluation of Coal Liquefaction Asphalt Modified Asphalt and Its Mixture. J. Zhengzhou Univ..

[3] Ji J., Yao H., Yang X., Xu Y., Suo Z., You Z. (2016). Performance Analysis of Direct Coal Liquefaction Residue (DCLR) and Trinidad Lake Asphalt (TLA) for the Purpose of Modifying Traditional Asphalt. Arab. J. Sci. Eng..

[4] Wang Z., Wang Z., Guo S., Sha C., Yang Y., Zhang M., Zhou X., Wang P. (2025). Influence of Coal Liquefaction Residues as Fine Aggregate Replacement on Asphalt Mixture Performance and Adsorption Characteristics. Sci. Rep..

[5] Zhao L., Wan J., Zheng G., Zhang Y. (2009). Rheological study of paving performance of high modulus asphalt binders. Build. Mater..

[6] Su Q., Liu C., Zheng Q., Li F., Xu B., Li H. (2023). Road Performance Test of Asphalt Mixture Modified by Coal Liquefaction Asphalt. China Highw..

[7] Ji J., Wang Z., Yao H., Wang D., Zhang R., Diab A., Dai Q. (2021). A numerical study on rutting behaviour of direct coal liquefaction residue modified asphalt mixture. Road Mater. Pavement Des..

[8] Ji J., Xu X., Xu Y., Wang Z., Wang J. (2021). Research on Performance of Direct Coal Liquefaction Residue Modified Asphalt Mortar. J. Fuel Chem. Technol..

[9] Wu Z., Guo X., Liu Y., Wang Y., Gao M., Yao C., Chang J., Zhou H. (2023). Construction of porous carbon for high-performance microwave absorbing: An efficient method to utilize direct liquefaction coal asphalt by-products. Diam. Relat. Mater..

[10] Zhang D., Luo R., Chen Y., Zhang S., Sheng Y. (2016). Performance Analysis of Coal Liquefaction Asphalt Modified Asphalt Based on Surface Free Energy. China J. Highw. Transp..

[11] Ji J., Yuan Z., Wei J., Suo Z., Xu Y., Li H., Shi Y. (2019). Improvements of low temperature properties of direct coal liquefaction residue modified asphalt. J. China Univ. Pet. Nat. Sci. Ed..

[12] Li Y., Kang X., Gao Q., Jia Y. (2022). Rheological properties of composite modified asphalt with direct coal liquefaction residues. Lect. Notes Civ. Eng..

[13] Gao Y., Hao Z., Zhang X. (2023). Interaction, rheological and physicochemical properties of emulsified asphalt binders with direct coal liquefaction residue based geopolymers. Constr. Build. Mater..

[14] Chen J. (2015). Modification of Petroleum Asphalt by Coal Liquefaction Asphalt Based on Aldehyde Crosslinking Agent. Master’s Thesis.

[15] Song Z., Sun M., Huang Y., Lv B., Su X., Zhong J., Zhao X., Ma X. (2017). Modified Asphalt with the Extract Fractions of Shenhua Direct Coal Liquefaction Residue. Chem. Ind. Eng. Prog..

[16] Ji J., Liu H., Yao H., You Z. (2024). Influence of compatibilization methods on the compatibility between asphalt and DCLR components with molecular dynamics (MD) method. Constr. Build. Mater..

[17] Zhao Y. (2015). Study on the Performance of Coal Liquefaction Asphalt Modified Asphalt and Its Mortar. Master’s Thesis.

[18] Ru Y. (2024). Development of Direct-Coal-Liquefaction-Residue-Based Composite Modifier for Direct Incorporation. Master’s Thesis.

[19] Seitenova G.Z., Dyussova R.M., Aspanbetov D.A., Jexembayeva A.Y., Korniejenko K., Aruova L., Sakanov D.K. (2025). Eco-Friendly Bitumen Composites with Polymer and Rubber Waste for Sustainable Construction. Buildings.

[20] Ji J., Wang Z., Li P., Zheng W., Xu X., Wang Z., Han B., Wei J., Li H. (2023). A review on direct coal liquefaction residue applied in asphalt pavements. J. Clean. Prod..

[21] Ji J., Suo Z., Zhang R., Li H., Han B., Wang J., You Z. (2021). Effect of physical hardening on low temperature performance of DCLR modified asphalt. Constr. Build. Mater..

[22] Liu Y., Wang Y., Ru Y., Li Y., Li Y., Gao Z. (2025). Rheological properties study of high-viscosity asphalt based on direct coal liquefaction residue. Front. Built Environ..

[23] Ji J., Liu H., Yao H., You Z. (2024). Influence of direct coal liquefaction residue (DCLR) on the rutting behavior of asphalt mixture with the discrete element method. Constr. Build. Mater..

[24] Ji J., Ma T., Zhang Z., Ling M., Xu X., Wei J. (2024). Evaluation of benzaldehyd and dioctyl phthalate modified direct coal liquefaction residue asphalt binder based on rheology and microscopic mechanisms. Clean. Mater..

[25] Gao Y., Zhang X., Jiang Z., Ding W., Miao D., Qiao Q. (2024). Utilization of direct coal liquefaction residue (DCLR) in recycled emulsified asphalt mixtures: A solution as geopolymer binder and filler materials. Constr. Build. Mater..

[26] Huang K. (2025). Study on Preparation and Performance of DCLR-Blended Asphalt via Extraction-Blending Process. Master’s Thesis.

[27] Li Y., Zhang X., Guo C., Gao J., Luo X., Zhang L., Yu H. (2026). Preparation of a direct addition composite modifier from direct coal liquefaction residue and its effect on the rheological properties of asphalt mastic. Front. Built Environ..

[28] Zhang T. (2023). Research Progress on Crumb Rubber-SBS Composite Modified Asphalt. Shanxi Traffic Sci. Technol..

[29] Wang Z. (2024). Study on Preparation and Pavement Performance of SBS-CR Composite Modified Asphalt. West. China Commun. Sci. Technol..

[30] Xie H. (2021). Study on Preparation and Performance of SBS/Crumb Rubber Composite Modified Asphalt (SBS/CRCMA). Master’s Thesis.

[31] Tan Y., Guo M., Cao L. (2013). Influence of Common Modifiers on Viscoelastic Properties of Asphalt. China J. Highw. Transp..

